# The Efficacy of Dural Puncture Epidural Performed by 27-gauge Whitacre Needle in Labour Epidural Analgesia: Randomized Single-Blinded Controlled Study

**DOI:** 10.4274/TJAR.2023.221085

**Published:** 2023-08-18

**Authors:** Iva Pažur, Ognjen Ožegić, Lada Lijović, Katarina Kličan Jaić, Maja Pešić

**Affiliations:** 1 Department of Anaesthesiology, Intensive Medicine and Pain Management, University Hospital Center Sestre Milosrdnice University, Zagreb, Croatia; 2 Department of Anaesthesia and Critical Care, Fra Mihovil Sučić Hospital, Livno, Bosnia and Herzegovina

**Keywords:** Dural puncture epidural, epidural analgesia, labour pain, neuraxial analgesia, obstetric anaesthesia

## Abstract

**Objective:**

Dural puncture epidural technique is refinement of standard epidural technique. Its goal is to overcome drawbacks of standard epidural. We assessed whether dural puncture epidural technique performed by 27-gauge spinal needle would provide higher quality of labour epidural analgesia by using 10 mL epidural bolus of 0.125% bupivacaine. Additionally, the impact of dural puncture epidural on epidural analgesia onset, course of labour and occurrence of maternal side effects was examined.

**Methods:**

We designed prospective, randomized, single-blind study. A total of 76 healthy nulliparous parturients were randomly allocated to dural puncture or standard epidural group. After identification of epidural space, spinal Whitacre needle was used for dural puncture. Intrathecal drug administration was omitted at that point. Both groups received a bolus of local anaesthetic mixture, followed by a continuous infusion of diluted local anaesthetic via epidural catheter. Pain was assessed by numeric pain rating scale. The number of top-ups and mode of delivery were recorded in both groups.

**Results:**

After 10 minutes, there was a statistically significant difference in numeric pain rating scale ≤3 reported (*P*=0.028), with 97.4% subjects in dural puncture epidural group achieving adequate analgesia after 10 minutes. There was no statistically significant difference in the number of additional boluses, time to delivery, Bromage scale achieved or maternal outcomes between groups.

**Conclusion:**

Dural puncture epidural technique appears to be effective in providing faster onset of epidural analgesia. However, the need for additional boluses remains unchanged. It can be safely used in obstetrics, without deleterious effect on the course of labour.

**Main Points**

• Dural puncture epidural technique enables faster onset of epidural labour analgesia.

• The clinical significance of dural puncture epidural technique in obstetrics remains equivocal.

• Dural puncture epidural technique could be helpful in conformation of epidural space.

• It is a safe technique for mother and child.

## Introduction

Dural puncture epidural (DPE) technique is a modification of combined spinal epidural, but it is devoid of intrathecal drug administration. It was introduced to obstetric anaesthesia clinical practice in a comparative study done by Chau et al.^1^ in 2017. Although epidural analgesia (EA) has been acknowledged as the most effective tool in relieving labour pain and is considered the gold standard in obstetric anaesthesia, the incidence of breakthrough pain under EA varies from 0.9 to 25% due to inconsistency in defining breakthrough pain.^2^ Slow onset of analgesia, block asymmetry, failed block, and insufficient analgesia in sensory distribution of sacral roots are the most common challenges in attainment of effective EA, especially during second labour stage.^2,3^ The aim of DPE in obstetric anaesthesia is to overcome these obstacles in providing satisfactory analgesia in parturients. Dural perforation by a spinal needle allows translocation of local anaesthetic (LA) from epidural space to cerebrospinal fluid (CSF) and greater caudal spread of medications, targeting sacral roots.^4,5^ Moreover, it represents additional confirmation that epidural space has been encountered. Notably, the risk of postdural puncture headache (PDPH) is being mitigated by using an atraumatic spinal needle.^6^ Primary aim of our study was to investigate whether DPE performed by 27 G spinal needle would provide higher quality of labour EA by using relatively small volume of epidural boluses, namely 10 mL of 0.125% bupivacaine. We hypothesized that the quality of EA would be improved by DPE technique. The secondary goal was to assess the impact of DPE on EA onset, course of labour and occurrence of maternal side effects related to neuraxial procedure.

## Methods

We performed a single centre, prospective, single-blinded, randomized controlled study to assess the effectiveness of DPE technique in comparison with standard epidural technique in labour analgesia. The study was conducted in Sestre Milosrdnice University Hospital Centre in Zagreb, Croatia from April 2021 to February 2022 after obtaining Institutional Ethics Board of Sestre Milosrdnice University Hospital approval on the 5^th^ of November 2020 (251-29-11-20-01-6). Trial registration was performed via Australia and New Zealand Clinical Trials Registry (ACTRN12622000976785). The study was conducted in accordance with the Helsinki Declaration. All participants recruited in the study provided written informed consent. The trial was blinded for patients but not for clinical investigators. Due to lack of anaesthesiologists in obstetric ward in our hospital we decided to conduct a single-blinded study with participants who were unaware of group allocation, while the members of anaesthesia team were informed about participant allocation. Seventy-six nulliparous parturients were randomly assigned using sealed opaque envelope technique to receive DPE or standard EA. Patients who met enrolment criteria were aged between 18 and 45 years in active labour with cervical dilatation from 3 to 6 cm at the moment of epidural insertion and with verbal numeric pain rating scale (NPRS) value greater than 3 during an uterine contraction. All parturients were at 38 to 42 weeks of gestation with healthy singleton pregnancy and foetal vertex position, classified as American Society of Anesthesiologists (ASA) physical status II. Exclusion criteria included hypertensive disorders in pregnancy (gestational hypertension, preeclampsia, eclampsia), placental disorders, contraindications for neuraxial anaesthesia, opioid addiction, allergy reaction on drugs used in the study, morbid obesity (body mass index >40 kg m^2-1^), patient refusal and unintended dural puncture by epidural needle. All procedures were performed by an anaesthesiologist experienced in the field of obstetric anaesthesia, defined as at least ten years of clinical experience.

The local clinical protocol was followed during procedure of epidural catheter placement. All participants were provided with peripheral venous access with 18-gauge cannula and maternal noninvasive blood pressure monitoring and pulse oximetry was initiated. A cardiotocography monitor was placed and monitored by obstetric personnel.

All participants received an intravenous co-loading of 500 mL of saline (0.9% NaCl) infusion before neuraxial procedure. Maternal hypotension was defined as a systolic blood pressure (SBP) less than 100 mmHg and/or a drop of more than 10% compared with the baseline preprocedural SBP.^7^ Treatment of hypotension consisted of placement in left or right decubital position and/or administration of vasopressor and additional bolus of fluids. For the purpose of this study we used individual epidural set and spinal kit. Lumbar epidural puncture was performed in a sitting position with an 18-gauge, 8 cm long Tuohy needle (Vygon, 5 rue Adeline - 95440 Ecouen, France)*,* at the level between third and fourth or fourth and fifth lumbar vertebra. Epidural space was identified by using loss of resistance technique with syringe containing 10 mL of saline. DPE parturients received a dural puncture by atraumatic Whitacre needle of 27-gauge and 12 mm of length (Vygon, 5 rue Adeline - 95440 Ecouen, France)*.* Spinal needle was inserted through epidural needle until free flow of CSF was obtained. Afterwards, the Whitacre needle was withdrawn and intrathecal drug administration was withheld. All epidural catheters of 19-gauge were introduced 5 cm into epidural space. Upon epidural catheter insertion and negative aspiration of blood or CSF, all participants were given a test dose (3 mL of 2% lidocaine) to rule out intrathecal epidural catheter placement. After the test dose came negative, EA was initiated with a 10 mL bolus of 0.125% bupivacaine administered over 5 minutes in both groups. We used fentanyl 1.5 mcg mL^-1^, as an analgesic adjuvant to LA. Analgesia was evaluated by verbal NPRS value between 0 and 10 during an uterine contraction (where 0 indicates no pain and 10 indicates the worst pain imaginable). After completion of epidural bolus (starting point), NPRS values were recorded at 5, 10 and 15 minutes. Further assessments of NPRS, level of sensory blockade and motor function were evaluated and recorded in 60 minute intervals until delivery or earlier on parturient’s request. If the pain was still present after 15 minutes had elapsed since the first bolus, the epidural catheter was withdrawn by 1 cm and a manual bolus was repeated. In case of inadequate response on second bolus or any other bolus given during labour, epidural catheter replacement was discussed with the parturient. Assessment of sensory block was done in each dermatomal level bilaterally for loss to blunt pinprick, cold and light touch sensation from cranial to caudal direction. Targeted upper dermatomal distribution of EA was bilateral Th10 level. The level of motor blockade was assessed by modified Bromage scale after bolus administration. Motor strength was assessed with a modified Bromage score (0 = full flexion of knees and ankles, 1 = partial flexion of knees, full flexion of ankles, 2 = inability to flex knees and partial flexion of ankles, and 3 = inability to flex knees and ankles, 4 = no movement). Examination of NPRS, sensory and motor block was done before and after administration of every additional bolus to ensure adequate analgesia with preserved motor function. The study team member who performed the assessment of sensory and motor function did not perform the block. The value of NPRS equal or less than 3 in the presence of an uterine contraction was defined as adequate analgesia. After the first bolus was given, continuous epidural infusion of 0.08% bupivacaine mixed with fentanyl 1.5 mcg mL^-1^ was immediately commenced at 8 mL per hour in both groups. Breakthrough pain was defined as NPRS >3 despite administration of continuous epidural infusion. The number of additional manual epidural boluses and NPRS values after bolus administration were recorded in both groups. Each bolus consisted of 10 mL of 0.125% bupivacaine mixed with fentanyl 1.5 mcg mL^-1^. Manual boluses were given by attending anaesthesiologist upon parturient’s request. Maximal allowed overall volume of LA mixture administered via epidural catheter was 25 mL per hour. Occurrence of maternal hypotension, pruritus, nausea and vomiting were recorded. Apgar scores were assessed by neonatologists after vaginal delivery and caesarean sections. Other collected data included record of side effects related to neuraxial procedure demanding intervention (hypotension, pruritus, nausea and vomiting), as well as failed conversion to epidural anaesthesia in case of emergency caesarean section. All participants were visited on postpartum day 1 by the member of study team who assessed the presence of headache, low back pain, nerve injury or any other complication related to neuraxial procedure.

### Statistical Analysis

Before the beginning of the study, power analysis showed that to achieve the power of 80% with a=0.05 and effect size of 0.5 a sufficient sample size would be n = 32. Normality of distribution of variables was tested using the Shapiro-Wilk test. The differences between quantitative variables were analysed using t-test for normally distributed variables, and Mann-Whitney U test was applied to variables that were not normally distributed. Continuous variables are shown as mean (standard deviation) or median (interquartile range). The differences between qualitative variables were compared using χ^2^ (chi-squared) test or Fisher’s exact test (for frequencies less than 5), where necessary. Values are presented as number and corresponding percentage, unless specified otherwise. All tests were two-tailed. *P* value of less than 0.05 was considered statistically significant. Statistical analysis was done using IBM SPSS 27.0.

## Results

**Figure 1:**
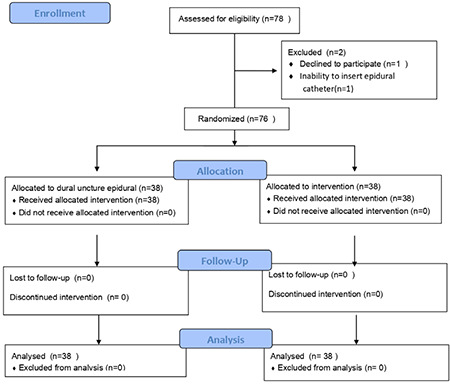
CONSORT flowchart of the patients progress throughout different phases of the study.

Seventy-eight parturients requesting EA were assessed for the eligibility for the study, two of them were excluded (Figure 1). A total of 76 patients were recruited. The groups were similar at baseline. Our results showed a significant difference in body mass index (BMI) between groups with mean value of BMI in DPE group 27.2 versus 29.7 in EA group (*P*=0.023). Moreover, we did not record cephalad spread of LA above targeted Th 10 level. Participants flow and baseline characteristics are summarized in Figure 1 and Table 1.

**Figure 2:**
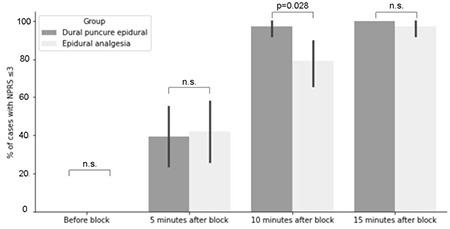
Epidural analgesia quality. NPRS was initially recorded 5, 10 and 15 min after first epidural bolus. Adequate NPRS value, defined as NPRS ≤3, was achieved in 97.4% of participants in subject group after 10 min following initial bolus. However, 5 min later there was no significant difference in NPRS value between groups. NPRS, numeric pain rating scale.

No significant difference in adequate analgesia achieved after 5 minutes was measured. After 10 minutes, there was statistically significant difference in NPRS score ≤3 achieved (Fisher’s exact test,* P*=0.028), with 97.4% subjects in DPE group achieving adequate analgesia after 10 minutes. However, the NPRS values were comparable between groups after 15 minutes (Figure 2). There was no statistically significant difference in additional boluses applied, time to delivery, Bromage scale achieved, mode of delivery and foetal outcomes between groups. Results are presented in Table 2. None of participants reported adverse reactions related to EA (PDPH, pruritus, hypotension, nausea and vomiting). The presence of CSF flow was successfully confirmed in all parturients in DPE group. There were no episodes of unintentional dural puncture by epidural needle. Also, there was no record of failed conversion to epidural anaesthesia in case of an emergency caesarean section.

## Discussion

There is paucity of data regarding the role of DPE in the field of obstetric anaesthesia, while the results of previous researches are often equivocal.^1,2,3,4,8,9,10^

In this randomized clinical trial we examined the effect of DPE in labouring women on EA onset time and quality of EA considering DPE influence on course of labour, mode of delivery and foetal outcome.

The principal finding of our study was that DPE performed by 27-gauge Whitacre needle provided faster onset of adequate labour EA compared to standard epidural technique. However, the number of additional boluses, time from first bolus to delivery, parturients’ motor function and Apgar scores did not differ significantly among groups. Our study also showed that the incidence of caesarean section and instrumented vaginal delivery were comparable between groups as well as the incidence of side effects.

Different sizes of spinal needles, LA concentrations and volumes of epidural catheter boluses and infusions were utilized in previous studies. In general, translocation of epidural medication in subarachnoid space is initiated by creating the dural hole. Intrathecal drug migration is influenced by the speed and the pressure of an injection applied via epidural catheter. Likewise, size of dural hole and volume of epidural bolus may also play important role in transmeningeal flux of anaesthetic drugs. Following this, it can be assumed that larger needle size and higher concentration of LA are related to faster analgesia onset and improved quality of EA but at the expense of compromised motor function and increased occurrence of side effects such as hypotension and pruritus.^2,3^ Potentially favourable effect of DPE in labour analgesia has been explained by improved coverage of sacral roots with LA offering better pain relief during second stage of labour. Ideal EA would provide satisfactory analgesia throughout the entire labour, while preserving motor function enabling smooth vaginal delivery.

According to literature, ropivacaine and bupivacaine are equally effective in regard to pain control. Although, ropivacaine showed higher specificity for sensory fibres due to its lower lipophilicity and slower spread to thick motor fibres in comparison with bupivacaine.^11^

However, our decision to use bupivacaine was based on the fact that bupivacaine in low doses (0.125% or less) has proved to be a safe LA in labour EA and is related to fewer additional top-ups in comparison with ropivacaine.^11,12,13^

Although epidural opioids are considered safe for mother and child, the effect of neuraxial opioids on foetal heart rate still remains a matter of debate.^14^ In our study fentanyl was administered as an analgesic adjuvant in slightly lower dose then in the majority of previous studies,^1,15,16^ namely 1.5 mcg per mL according to our local institutional protocol. We chose this approach trying to minimize the risk of foetal bradycardia and maternal pruritus.

There is a lack of consensus in literature whether to give test dose in labour analgesia or not. Moreover, there is heterogeneity in concentration, volume and type of LA used as a test dose.^17^ In the study by Yadav et al.^15^ after identification of epidural space, test dose was not administered. Instead, upon epidural catheter placement, epidural bolus of LA was immediately given in fractionated manner, using small aliquots of low concentration of LA as an alternative to traditional test dose. In our study, a test dose consisting of 3 mL of 2% lidocaine without adrenaline, was given prior the epidural bolus as part of local institutional protocol. Administration of test dose was devoid of any complications in terms of intravascular injection, high spinal or motor weakness of lower extremities.

To the best of our knowledge, in comparison with previous studies in the field of labour DPE, we used the smallest volume of bupivacaine as an induction dose for labour EA (namely, 10 mL of 0.125% bupivacaine). Unlike the majority of published studies, in our research larger volume of additional top-ups were deploy. The rationale for this is as the labour progresses and second labour stage begins, larger volume of single top-up is necessary to enable spread of LA around sacral roots which innervate birth canal. In contrast to previous studies which utilized patient controlled EA (PCEA), we decided to deploy a combination of manually given boluses on parturient’s request with continuous infusion of LA via epidural catheter. We opted for this approach because PCEA devices are not available in our facility.

Our choice to use 27-gauge Whitacre needle was based on results obtained in the study by Contreras et al.^18^ They showed that DPE with 25-gauge pencil point needle enabled 1.6 min shorter onset of EA in comparison with 27-gauge pencil point needle. Nonetheless, authors concluded that this result was of vague clinical relevance.^18^

Moreover, young pregnant woman are at very high risk for development of PDPH which is associated with long-term morbidity (persistent headache and backache) and some possibly life-threating complications (cerebral haemorrhage, dural sinus thrombosis).^19,20^ Considering these facts, we decided to test the efficacy of 27-gauge Whitacre needle in DPE procedure as more favourable option in comparison with larger spinal needle size.

Recent studies obtained faster onset of adequate EA in DPE group regardless of spinal needle size. Concerning request for additional top-ups and course of labour, most of authors showed no significant difference among DPE and standard epidural. Song et al.^#*#ref21#*^# found that a combination of DPE with programmed intermittent epidural boluses showed most favourable results regarding EA onset, number of additional boluses and LA consumption.

According to Yadav et al.,^15^ higher quality of EA was reported in DPE group. The number of parturients achieving adequate analgesia in 5 and 10 min was significantly higher in DPE group than in control group.^15^ Although we used similar protocol, in our study significantly higher number of parturients reported adequate analgesia in DPE group only 10 min after first epidural bolus. The possible explanation could lie in the fact that study by Yadav et al.^15^ and our trial were conducted on different type of population (Indian versus European). Also, there was difference in LAs (ropivacaine versus bupivacaine) as well as in mode of LA delivery (solely epidural boluses versus epidural boluses in addition to continuous epidural infusion of LA and opioid).

Thomas et al.^16^ pointed out that when no CSF returned from the 27-gauge spinal needle after an attempted dural puncture, the catheter inserted into the epidural space might be less functional with a higher replacement rate. Nevertheless, EA quality was not improved in DPE group.^16^ Potential beneficial effect of DPE was examined in population of obese parturients (BMI >35 kg m^2-1^) who are at higher risk for epidural failure, so the dural puncture technique may have particular utility in this population. Authors showed no additional benefit of DPE to support routine use of DPE in obese parturients.^22^

Cappiello et al.^23^ demonstrated improved quality and onset of EA with DPE with 25-gauge spinal needle but at the cost of increased incidence of instrumented vaginal delivery. This might be explained by using larger spinal needle size and higher concentration of LA (0.25% bupivacaine bolus followed by 0.125% bupivacaine epidural infusion).^23^ Chau et al.^1^ also utilized 25-gauge needle in their study, comparing efficiency of DPE with epidural and combined spinal epidural techniques (CSE). Contrary to majority of studies in this field, they found no difference in analgesia onset between DPE and standard epidural technique, while most rapid onset of analgesia was recorded in CSE group. This could be attributed to the large volume of initial epidural bolus (20 mL of 0.125% bupivacaine) applied in both groups, DPE and standard epidural technique. Parturients’ motor function remained preserved probably due to lower LA concentration (0.125% bupivacaine bolus and infusion). Notably, DPE provided improved block quality in comparison with standard epidural and fewer maternal and foetal side effects than the CSE technique. In our study, we did not find increased incidence of side effects related to DPE.

The reason why we did not achieve more beneficial effect of DPE might be explained by the fact that we used low concentration of LA mixture, along with relatively small volume of LA bolus in comparison to some studies in which boluses up to 20 mL were utilized.^1^ We decided for aforementioned approach trying not to jeopardize the obstetric outcome by provoking assisted vaginal delivery.^24,25^

### Study Limitations

This study does have some limitations. Exact time of EA onset is sometimes difficult to establish considering that we assessed the pain during uterine contractions which could occur in irregular intervals.^26^ Moreover, in our opinion, it is difficult to precisely assess the pain by the conventional diagnostic tools (visual analogue scale, NPRS, etc.) in labouring women population. A second limitation is the lack of blinding. However, all NPRS assessments were done by a member of anaesthesia team not directly involved in the epidural catheter placement and participants were unaware of group allocation.

In our opinion, DPE provides faster onset of EA regardless of the spinal needle size. We consider DPE as a valuable tool in confirmation of subarachnoid space, which strongly indicates that the tip of epidural needle is in epidural space. What remains to be established is the optimal combination of spinal needle size, dosing regimen of epidural boluses and the concentration of LA.

## Conclusion

In conclusion, DPE provides faster onset of adequate labour EA by using 27-gauge Whitacre needle. However, the clinical importance of this finding is uncertain. We deem that quality of labour EA is not improved, because the request for additional epidural boluses alongside continuous epidural infusion of a LA and an opioid remains unchanged compared to standard epidural technique. It possesses no adverse effect on the course of labour.

## Figures and Tables

**Table 1:**
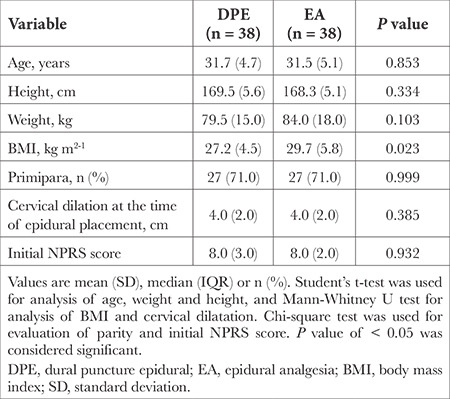
Subject Baseline Characteristics

**Table 2:**
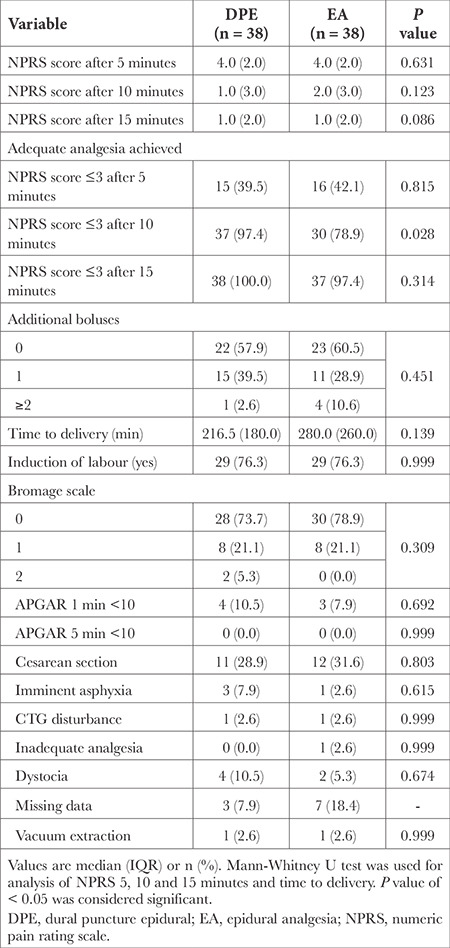
Neuraxial Block Quality and Maternal Outcomes
